# Behavioral and electrophysiological evaluation of the anesthetic therapeutic window of thyme essential oil in tambaqui (*Colossoma macropomum*)

**DOI:** 10.1007/s10695-026-01783-0

**Published:** 2026-09-07

**Authors:** Maria Juliana da Luz Froz, Axell Lins, Rômulo Augusto Faio Farias, Diogo Macola Felix, Eduardo Machado da Silva Zamian, Eva Vitoria Ferreira Viana, Xaiane Mikelly Baia Viana, Moisés Hamoy

**Affiliations:** 1 Laboratory of Pharmacology and Toxicology of Natural Products, Federal University of Pará (UFPA), Belém, Brazil; 2 Postgraduate Program in Pharmaceutical Science, Faculty of Pharmacy, Federal University of Pará, Belém, Brazil

**Keywords:** *Thymus vulgaris*, Tambaqui, Therapeutic window, Electrocorticography, Electrocardiography

## Abstract

This study aimed to determine the anesthetic therapeutic window of *Thymus vulgaris* L. essential oil (TVEO) in juvenile *Colossoma macropomum* (tambaqui), through the integrated analysis of behavioral parameters, electrocardiographic recordings, opercular activity during the induction, and recovery periods of anesthesia, as well as the measurement of blood glucose levels in the animals after the anesthetic procedure. The fish were divided into the control, vehicle, and TVEO-treated groups at concentrations of 18.6 mg. L⁻^1^, 27.9 mg. L⁻^1^, 37.2 mg. L⁻^1^, and 46.5 mg. L⁻^1^, administered by immersion baths. Anesthetic efficacy was evaluated by means of induction time, loss of righting response, and recovery time. Electrocardiographic recordings were used to monitor heart rate and rhythm, allowing the analysis of possible pharmacological effects on cardiac electrical conduction. A concentration of 18.6 mg. L⁻^1^ promoted mild anesthesia without loss of postural response or relevant cardiovascular changes. Concentrations of 27.9 mg. L⁻^1^ and 37.2 mg. L⁻^1^ induced effective anesthesia, with rapid induction, adequate recovery, and electrocardiographic stability, characterizing the main therapeutic window. The concentration of 46.5 mg. L⁻^1^ resulted in transient cardiovascular depression, evidenced by a reduction in heart rate, suggesting autonomic modulation and possible interaction with cardiac ion channels. *Thymus vulgaris* essential oil exhibits concentration-dependent anesthetic action *in C. macropomum*, with concentrations of 27.9, 37.2, and 46.5 mg. L⁻^1^ representing the window for anesthesia in moderate to deep planes. The findings indicate the involvement of central and cardiovascular pharmacological mechanisms, reinforcing the potential of TVEO as a natural alternative to synthetic anesthetics in fish.

## Introduction

Professionalization and animal welfare in aquaculture has been promoting the use of anesthetics in aquatic animals for experimental protocols, such as biometrics, transport, collection of biological samples, invasive procedures, and even as experimental models (Supriya et al. 2022). However, these activities frequently trigger physiological stress responses, characterized by behavioral, metabolic, respiratory, and cardiovascular changes that can disrupt the organism’s homeostasis (Roumbedakis et al. 2020). These responses can compromise animal welfare and increase mortality or interfere with the reliability of experimental data, making the use of anesthetic agents fundamental for chemical restraint with less adverse reactions such as stress (Park et al. 2017).

Synthetic anesthetics like benzocaine, and tricaine methanesulfonate (MS-222) and natural anesthetics such as eugenol are widely used; however, these compounds had potential adverse effects on heart and respiratory rate, seizure activity, and serious injuries (Félix et al. 2025; Mocho et al. 2022). Given these limitations, there is growing interest in natural anesthetics, particularly essential oils from medicinal plants. Their diverse secondary metabolites provide effective sedation while offering aquaculture farmers a more accessible, eco-friendly, and acceptable alternative. (Silva et al. 2020; Baesso et al. 2024).

In this context, the essential oil of *Thymus vulgaris* L., belonging to the Lamiaceae family, has stood out for its multiple pharmacological properties, widely described in the literature, including antimicrobial, antioxidant, anti-inflammatory, and neuroprotective activities (Galovičová et al. 2021; Patil et al. 2021; Hosseinzadeh et al. 2015). Its main constituents, such as thymol, p-cymene, and carvacrol, demonstrate the ability to modulate ion channels, neurotransmitters, and inflammatory pathways, being associated with sedative, anxiolytic, and central nervous system depressant effects, which suggests its potential use as an anesthetic agent in aquatic organisms (Wang et al. 2020; Nadi et al. 2023; Zhao et al. 2024).

Amazonian fish species of economic and ecological importance, such as tambaqui (*Colossoma macropomum*), widely cultivated in Brazil, require specific studies that consider their physiological particularities and their sensitivity to anesthetic agents with practice that makes the procedures safe (Nascimento et al. 2024; Val & Oliveira 2021). Anesthetics are currently evaluated through the behavior of induction of anesthetic plans, taking into account the response to painful stimuli and drug reversibility. However, the monitoring of cardiac and respiratory function by means of electrophysiology has been increasingly used to evaluate the safety of anesthetic drugs in fish. Thus, changes in heart rate, electrocardiographic intervals, and opercular activity may indicate adverse effects or safety limits of the anesthetic, being fundamental tools for evaluating the viability of new anesthetic substances in fish (Hamoy et al. 2023; Vilhena et al. 2022).

Thus, the present study aimed to determine the anesthetic therapeutic window of *Thymus vulgaris* L. essential oil in juveniles of *Colossoma macropomum*, through the integrated analysis of behavioral parameters, electrocardiographic recordings, opercular activity during the periods of induction, and anesthetic recovery, as well as the measurement of the animals’ blood glucose after the anesthetic procedure. Thus, the results of this work aim to contribute to the knowledge and safe use of natural anesthetics in aquaculture and animal experimentation.

## Material and methods

### Experimental animals

The individuals used were 108 juveniles of tambaqui, *Colossoma macropomum*, of both sexes, weighing on average 23 ± 3.2 g. The project was approved by the Ethics Committee on the Use of Animals of the Federal University of Pará (CEUA/UFPA), under protocol number CEUA no. 1777260924. The animals were kept in five aquariums with a capacity of 200 L in the Experimental Vivarium of the Laboratory of Pharmacology and Toxicology of Natural Products of the Federal University of Pará (ICB/UFPA) in an environment with controlled temperature (25 to 27 °C) and photoperiod 12 h of light and 12 h of dark, feeding twice a day with commercial feed (32% protein). The aquariums were daily siphoned to remove uneaten food and feces; the water was partially renewed (approximately 30% of the aquarium volume) with filtered water from the same source. During acclimatization (10 days), water quality variables such as water temperature (°C) (26.4 °C) and hydrogen potential (pH) (pH = 7.7) were monitored.

### Organoleptic properties and chromatographic analysis

**Fig. 1:**
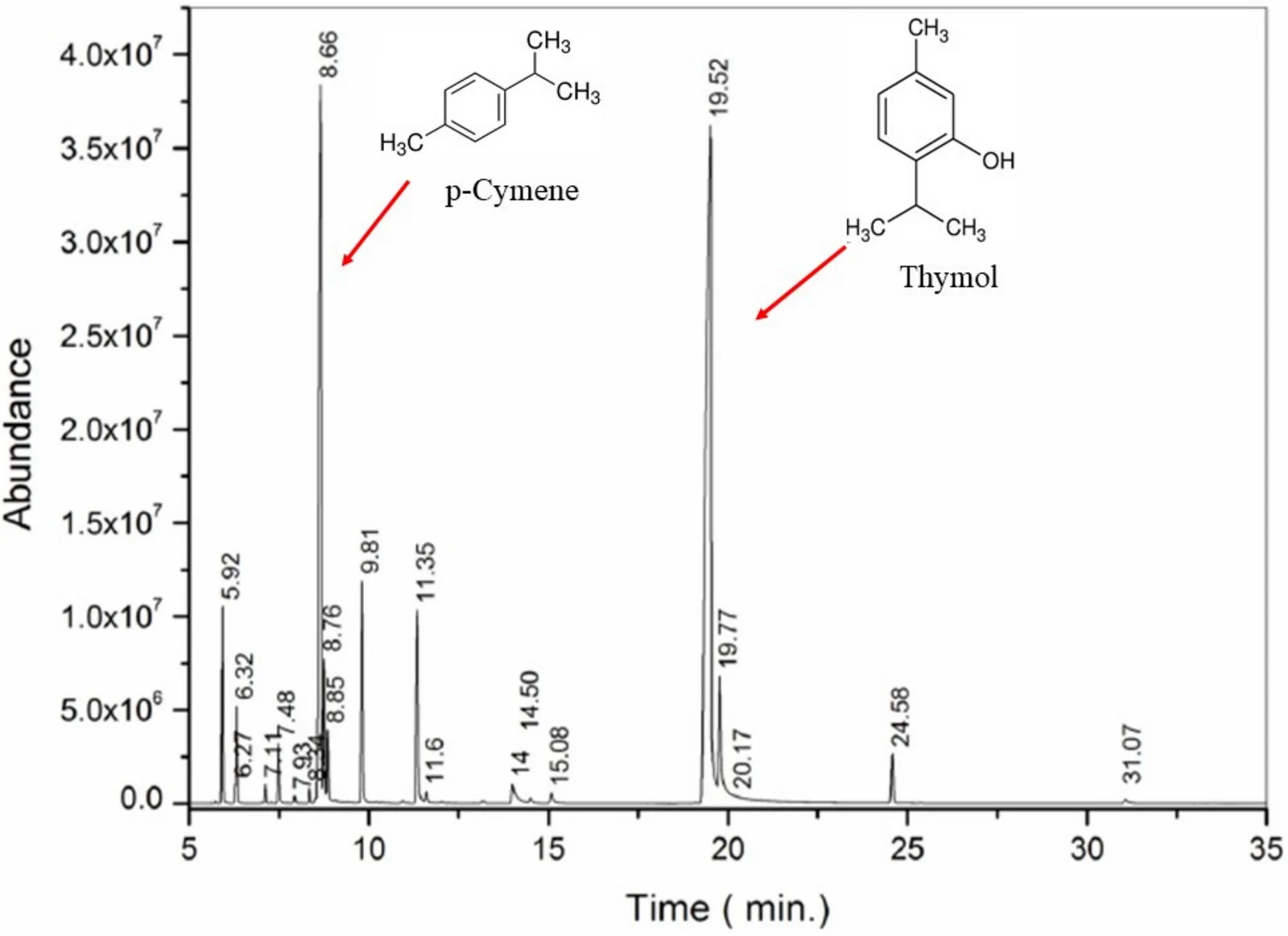
Chromatogram of the essential oil sample of *Thymus vulgaris* L. (TVEO), gas chromatography–mass spectrum GC–MS. Technology Center of the Federal University of Santa Catarina (SC)—Brazil

Essential oil *Thymus vulgaris* L. (TVEO) was extracted by distillation and steam drag and the method of analysis was high gas chromatography: column: HP-5 30 m × 0.32 mm × 0.25 μm (AGILENT). Temp.: Column: 70 °C (0 min), 3 °C/min to 250 °C. Injector: 250 °C Division: 1/50. FID detector: 260 °C. injection vol.: 1 μL (1% in chloroform). The phytoconstituents were analyzed by the Federal University of Santa Catarina–Technology Center–Department of Chemical Engineering and Food Engineering with the identification code 23/063, date of the exam 01.09.2023. Density of 0.930 g/cm^3^ with an intense and herbal aroma with a light-yellow color. Substances identified are described in Table 1, with the major components being thymol (46.40%), p-cymene (22.75%), and carvacrol (6.58%) (Fig. 1).

**Table 1:** Composition of essential oil of *Thymus vulgaris* L. (TVEO) used in the treatments in immersion bath in *Colossoma macropomum*

Molecular formula	TR	Identified substance	%
C10H16	5.92	α-Pinene	3.10
C10H16	6.32	Camphene	1,84
C10H16	7.11	β-Pinene	0.33
C10H16	7.48	Myrcene	1.17
C10H16	7.93	α-Phellandrene	0.17
C10H16	8.34	α-Terpinene	0.30
C10H14	8.66	p-Cymene	22.75
C10H16	8.76	D-Limonene	2.53
C10H18	8.85	Eucalyptol	1.46
C10H16	9.81	γ-Terpinene	4.59
C10H18O	11.35	Linalool	4.68
C10H16O	11.60	α-Thujone	0.31
C10H18O	14.00	Endo-Bomeol	1.11
C10H18O	14.50	Terpinen-4-ol	0.24
C10H18O	15.09	α-Terpineol	0.44
C10H14O	19.52	Thymol	46.40
C10H14O	19.78	Carvacrol	6.58
C15H24	24.58	Caryophyllene	1.22
C15H24O	31.07	Caryophyllene oxide	0.23
		Minority compounds	0.29
		Unidentified compounds	0.25

### Experimental design

## Thymus vulgaris L. essential oil experiment (TVEO)

The tambaqui juveniles were arbitrarily distributed in the following treatments: a) control; b) vehicle group (fish exposed to immersion bath: 1 mL of absolute ethyl alcohol in 1 L aquarium water tank; c) fish treated with TVEO 18.6 mg. L^−1^; d) 27.9 mg. L^−1^; e) 37.2 mg. L^−1^; and f) 46.5 mg. L^−1^. All fish were exposed to anesthetic induction in an immersion bath for a period of 5 min, and for anesthetic recovery, it was observed for 5 min in water from the aquarium without TVEO. For each record, one *n* = 9/treatment (immersion bath with TVEO and recovery after immersion bath) was used in a total of 108 juveniles of *Colossoma macropomum*.

## Experiment 1: analysis of the characteristic behavior of induction and anesthetic recovery

Considering the contact time in immersion bath with TVEO at the concentrations: a) control; b) vehicle group; c) TVEO 18.6 mg. L^−1^; d) TVEO 27,9 mg. L^−1^; e) TVEO 37.2 mg. L^−1^, and f) EOTV 46.5 mg. L^−1^ (*n* = 9/treatment), the latency time for the behavior of loss of posture reflex and immobility was evaluated. Subsequently, the animals were removed from contact with TVEO, where the latency for recovery of the posture reflex was evaluated.

## Experiment 2: analysis of blood glucose after treatment

Thirty minutes after the end of treatment with TVEO in the groups, blood was collected from the caudal vessels of the *Colossoma macropomum*. The test was done with a drop of blood through an Accu-Chek Active (mg/dL) blood glucose meter.

## Experiment 3: electrocardiogram (ECG) analysis and recording of opercular activity during induction and recovery

For the analysis of the cardiac function recording, the groups were divided: a) control group; b) vehicle group; c) group treated with TVEO 18.6 mg. L^−1^; d) 27.9 mg. L^−1^; e) 37.2 mg. L^−1^; and f) 46.5 mg. L^−1^ (*n* = 9, total of 54 animals). For this, electrodes were made in 925 silver with a diameter of 0.3 mm and a length of 15 mm; later, they were isolated with liquid insulator (Quimatic). The electrodes were made in a nonconjugated way. The reference electrode was positioned in the ventral part of the right opercular opening (negative pole), and the recording electrode was fixed in the ventral portion of the left operculum (positive pole). The electrodes picked up the signal in lead D1. The electrodes were connected to a high-impedance amplifier (Grass Technologies, Model P511) for the acquisition of electrocardiographic recordings. Enabling the analysis of heart rate (bpm), duration of the QRS complex (ms), R-R (ms), P-Q (ms), and S-T (ms) intervals.

To acquire the opercular activity record, 925 silver electrodes were made with a diameter of 0.5 mm and a length of 15 mm. The electrodes were conjugated at a distance of 6 mm and isolated with liquid insulator (Quimatic). The positioning of the electrode fitting to record the opercular beat was made in the central part of the right opercular opening. During the recordings, the frequency (opercular movements per minute) and the power of the opercular beat (mV) were evaluated (^2^/Hz).

### Recording and analysis of records

The electrodes were connected to a digital data acquisition system through a high-impedance input differential amplifier (Grass Technologies, Model P511), tuned with filtering of 0.3 and 300 Hz, with 2000X amplification and monitored with an oscilloscope (ProteK, Model 6510). The records were continuously digitized at a rate of 1 kHz on a computer equipped with a data acquisition board (National Instruments, Austin, TX), and were stored on a hard disk and later processed through specialized software (LabVIEW express).

The analysis of the acquired signals was possible with the help of a tool built in the Python programming language. The Numpy and Scipy libraries were used for mathematical processing and the Matplolib library was used for the elaboration of the graphs. The graphical interface was developed using the PyQt4 library (Hamoy et al. 2023).

### Statistical analysis

After evaluating compliance with the assumptions of normality and homogeneity of variances, using the Kruskal–Wallis and Levene tests, respectively, the comparisons of the mean values were made using two-way ANOVA, followed by Tukey’s test. The GraphPad Prism software 8 was used for the analyses and a value of ^*^p < 0.05 was considered statistically significant in all cases.

## Results

**Fig. 2:**
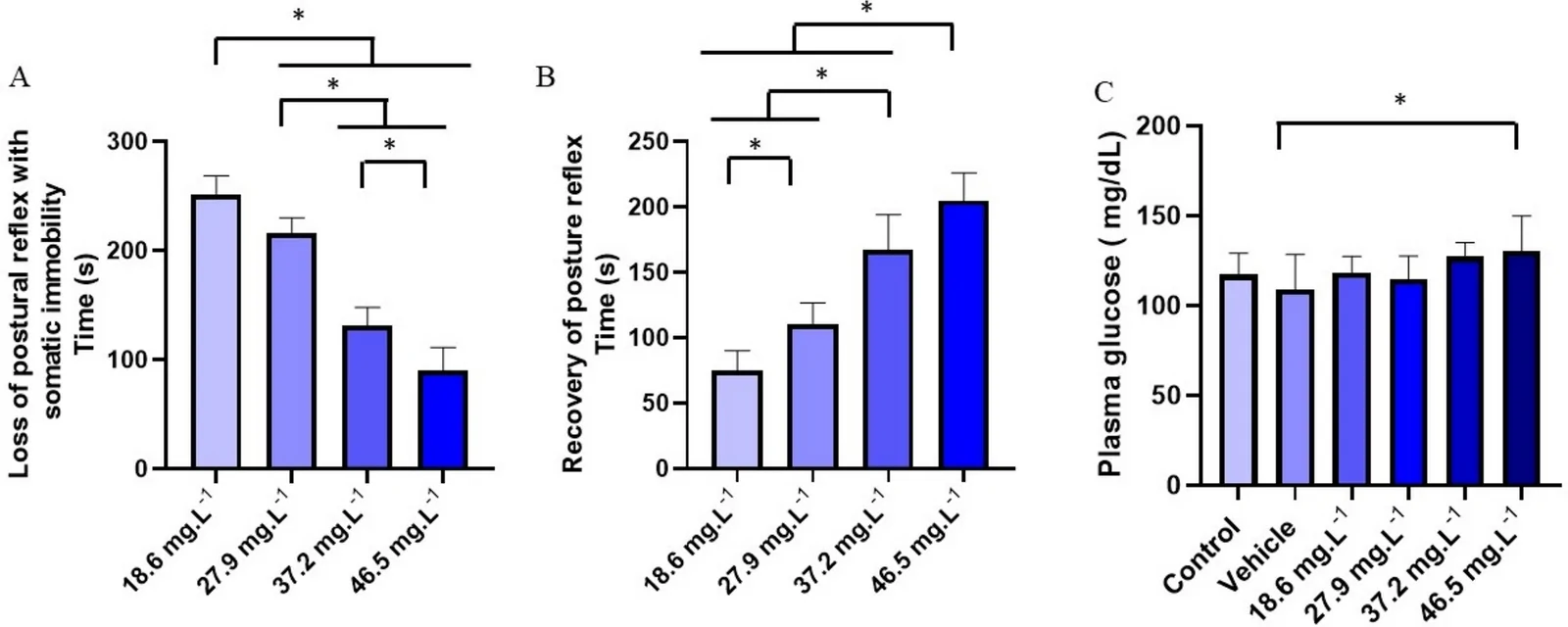
Average latencies in seconds for loss of postural reflex during immersion baths with different TVEO treatments (**A**). Recovery of postural reflex after treatment with different concentrations of TVEO (**B**) and average blood glucose in juveniles of *Colossoma macropomum*, thirty minutes after treatment at different concentrations of TVEO. (ANOVA followed by Tukey’s test; ^*^p < 0.05)

During the treatment with TVEO, behavioral changes were observed during anesthetic induction, the main one being the loss of postural reflex in the fish and immobility; in this case, the treatments with higher concentrations presented a shorter latency period for the appearance of the behavior. Treatment with 18.6 mg. L^−1^ showed mean loss of posture reflex at 251.6 ± 17.36 s, which was higher than the other groups. The group treated with 27.9 mg. L^−1^ had a mean latency of 216.7 ± 13.67 s, 37.2 mg. L^−1^ (131.8 ± 16.23 s) and 46.5 mg. L^−1^ (89.78 ± 21.68 s) were different, demonstrating that a small variation in the concentration of the treatment led to a rapid deepening of the anesthetic (Fig. 2A).

Recovery of the postural reflex in the group treated with 18.6 mg. L^−1^ of TVEO had a latency of 75.11 ± 15.19 s, which was lower than the other groups: 27.9 mg. L^−1^ (110.1 ± 16.73 s), 37.2 mg. L^−1^ (166.9 ± 27.35 s), and 46.5 mg. L^−1^ (205.0 ± 21.07 f.). All groups showed latency for recovery depending on the concentration used, demonstrating reversibility of the effect more slowly for the groups that received higher concentrations (Fig. 2B).

The assessment of plasma glucose thirty minutes after treatment with TVEO, the control group (117.1 ± 11.95 mg/dL) was similar to the vehicle group (*p* = 0.815) and to all groups treated with TVEO (*p* = 0.367). The vehicle group (108.8 ± 19.70 mg/dL) was inferior to the treated group with 46.5 mg. L^−1^ (*p* = 0.026) (Fig. 2C).

**Fig. 3:**
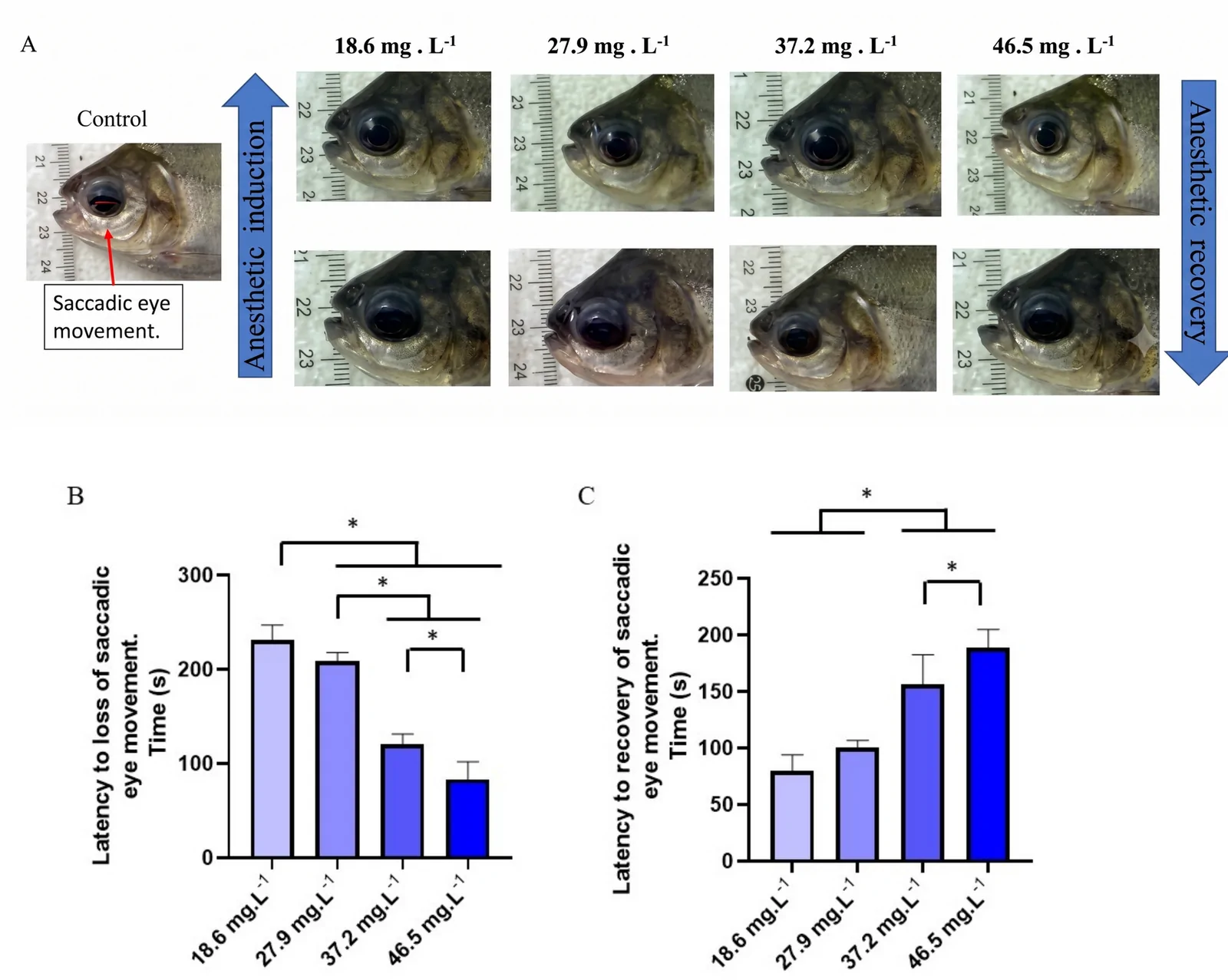
It presents a photographic record of the ocular saccadic movement of the tambaqui during anesthesia with TVEO, during anesthetic induction there is loss of saccadic movement of the eyes, during the recovery period ocular paralysis is reversed (**A**); the graphs demonstrate the average latencies (seconds) for loss of saccadic eye movement during immersion baths with different concentrations of TVEO (**B**). Recovery of saccadic movement of the eyes after contact with different concentrations of TVEO (C). (ANOVA followed by Tukey’s test; ^*^p < 0.05)

The behavioral analysis showed that the treatment with TVEO caused paralysis of the eye movements in the tambaqui during anesthesia (Fig. 3A); its paralysis indicates deepening of the anesthesia with TVEO. The latency to eye movement paralysis for the group treated in immersion bath with 18.6 mg. L^−1^ TVEO, with a mean latency of 230.8 ± 16.40 s, was higher than the other groups: 27.9 mg. L^−1^ (208.8 ± 9.25 s), 37.2 mg. L^−1^ (120.3 ± 18.56 s), and 46.5 mg. L^−1^ (83.67 ± 18.56 s). Thus, all groups showed differences, as the latency for loss of eye movements was lower for the groups treated with the highest concentrations (Fig. 3B).

The recovery of eye movement after the 5-min contact time in the TVEO immersion bath (Fig. 3C) for the group treated with 18.6 mg. L^−1^ had a mean of 80.22 ± 14.13 s, similar to the treated group with 27.9 mg. L^−1^ (*p* = 0.081). However, 37.2 mg was lower than the other treated groups 37.2 mg. L^−1^ (156.6 ± 26.21 s) and 46.5 mg. L^−1^ (189.3 ± 15.63 s). The group treated with 46.5 mg. L^−1^ was superior to the treated group at 37.2 mg. L^−1^ (Fig. 3 C).

**Fig. 4:**
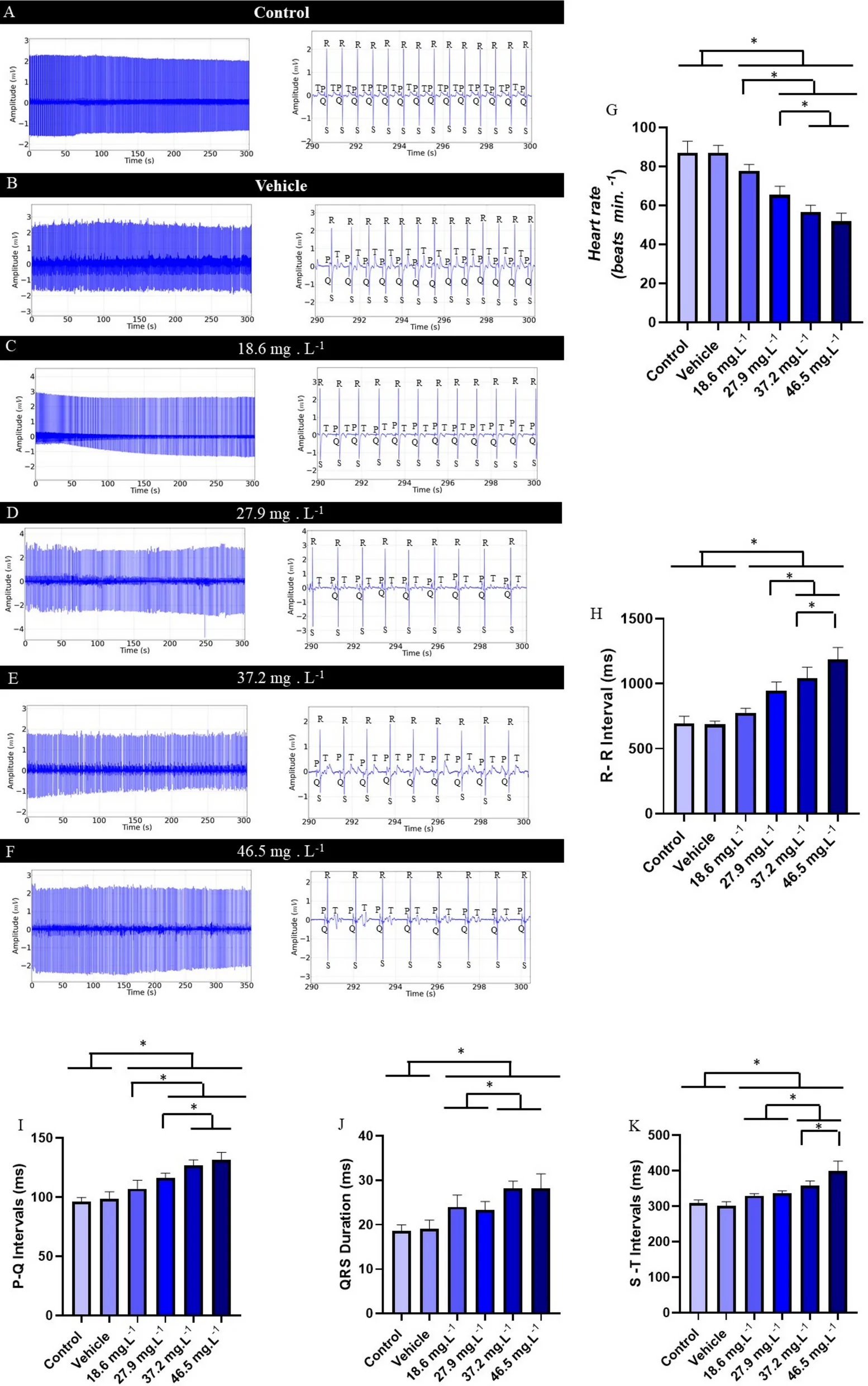
Electrocardiographic recordings demonstrating cardiac activity in juveniles of *Colossoma macropomum* (left), amplification of the 10 s at the end of the 5-min recording (290–300 s) (center), demonstrating the graphoelements indicating the P wave, QRS complex and T wave for the following groups: control group (**A**), vehicle group (**B**), group treated with 18.6 mg. L^−1^ (**C**), 27.9 mg. L^−1^ (**D**), 37.2 mg. L^−1^ (E), and 46.5 mg. L.^−1^ (**F**). Analysis of the data shown in graphs of heart rate (bpm) (**G**), RR interval (*ms*) (**H**), PQ interval (ms) (**I**), QRS duration (ms) (**J**), and ST Interval (ms) (**K**). (ANOVA followed by Tukey’s test; *p < 0.05; *n* = 9)

The normal electrocardiogram of *Colossoma macropomum* showed sinus rhythm and mean frequency of 86.89 ± 6.09 bpm, the graphoelements were identified in the P wave the QRS complex and where T (Fig. 4A). Cardiac activity in the control group was similar to that of the vehicle group (*p* = 0.999) (Fig. 4B). From the 10-s amplification, the intervals during treatment with TVEO were evaluated (Fig. 4C–F).

During treatment in immersion bath with 18.6 mg. L^−1^, the animals showed a 10.71% reduction in heart rate in relation to the control group (Fig. 4C), for the group treated with 27.9 mg. L^−1^ TVEO decreased by 24.87% (Fig. 4D), group treated with 37.2 mg. L^−1^ showed a decrease of 34.77% (Fig. 4E) and 46.5 mg. L^−1^ the reduction was 40.40% (Fig. 4F). The treated fish showed bradycardia in sinus rhythm, which was intensified by the increase in concentration, but did not present arrhythmias during anesthesia.

ECG recording demonstrated sinus bradycardia during treatment with increasing concentrations of TVEO. The control had a mean of 86.89 ± 6.09 bpm, similar to the vehicle (*p* = 0.999). However, they were superior to the groups treated with TVEO. The group treated with 18.6 mg. L^−1^, with a mean of (77.56 ± 3.43 bpm) was higher than the other treated groups. The group treated with 46.5 and 37.2 mg. L^−1^ (51.78 ± 4.17 bpm) was similar to the group treated at 37.2 mg. L^−1^ (*p* = 0.179). However, they were lower than the other groups (Fig. 4 G).

The mean RR interval for the control group was 693.7 ± 55.66 ms was similar to the mean RR interval for the vehicle group (*p* = 0.999) and for the group treated with 18.6 mg. L^−1^ (*p* = 0.108), however, were lower than the other groups treated. The group treated with 46.5 mg. L^−1^ had a mean RR interval of 1134 ± 92.07 ms, was higher than in all groups (Fig. 4H).

The mean PQ interval for the control group was 96.22 ± 3.56 ms, similar to the vehicle group (*p* = 0.930). The groups treated with 46.5 mg. L^−1^ (131.7 ± 6.24 ms) was similar to the 37.2 mg. L^−1^–treated group (*p* = 0.385) but were higher than the other groups (Fig. 4I).

The mean duration of the QRS complex for the control group (18.56 ± 1.42 ms) was similar to that of the vehicle group (*p* = 0.994), but was lower than the other treated groups. The group treated with 18.6 mg. L^−1^ (24.0 ± 2.26 ms) was similar to the treated group at 27.9 mg. L^−1^ (*p* = 0.987). The group treated with 46.5 mg. L^−1^ (28.22 ± 3.23 ms) was similar to the treated group at 37.2 mg. L^−1^ (*p* = 0.999), however, were higher than the other groups (Fig. 4 J).

For the control group, the mean ST interval was 308.8 ± 8.85 ms was similar to the vehicle group (_p_ = 0.883), but was lower than the other groups. The group treated with 18.6 mg. L^−1^ (329.8 ± 5.91 ms) was similar to the group treated with 27.9 mg. L^−1^ (*p* = 0.923). The group treated with 46.5 mg. L^−1^ (399.0 ± 28.10 ms) was higher than the other treated groups (Fig. 4 K).

**Fig. 5:**
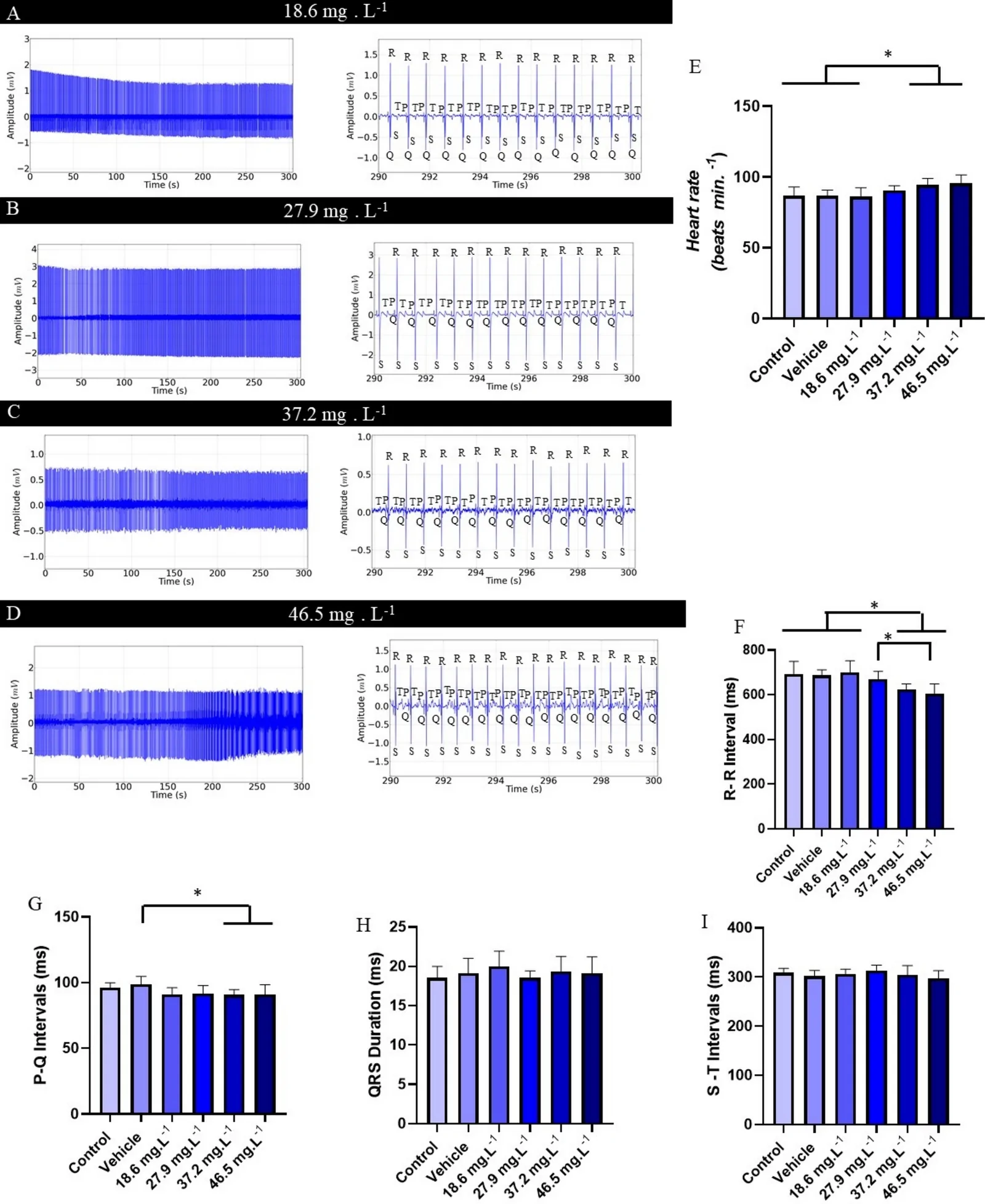
Cardiac activity in juveniles of *Colossoma macropomum* after immersion bath (left). Amplification of the recording in the last 10 s (290–300 s), with identification of cardiac deflagrations (center), in the recovery period after immersion bath with the following concentrations of TVEO at the following concentrations: 18.6 mg. L^−1^ (**A**), 27.9 mg. L^−1^ (**B**), 37.2 mg. L^−1^ (**C**), and 46.5 mg. L^−1^ (**D**). Mean values of cardiac parameters during recovery from treatment: Values of mean heart rate (bpm) (**E**), RR interval (mV) (**F**), *P*-*Q* intervals (ms) (**G**), duration of QRS complex (ms) (**H**), and ST interval (ms) (**I**). (ANOVA followed by Tukey’s test; ^*^p < 0.05; *n* = 9)

During recovery from anesthesia induced by TVEO, reversibility of electrocardiographic changes was observed (Figs. 5A–D). However, the recovery happened fully for all treated groups.

Recovery of cardiac activity in the group treated with 18.6 mg. L^−1^, 27.9 mg. L^−1^, 37.2 mg. L^−1^, and 46.5 mg. L^−1^ in relation to the control group, it was 100% (Fig. 5A–D).

During the recovery period, the control group had a mean heart rate of 86.89 ± 6.09 bpm, which was similar to the vehicle group (*p* = 0.999), the group treated with 18.6 mg. L^−1^ (*p* = 0.999) and group treated with 27.9 mg. L^−1^ (*p* = 0.636). However, they were inferior to the group treated with 37.2 mg. L^−1^ and 46.5 mg. L^−1^ (Fig. 5E).

The mean RR interval during recovery for the control group was 693.7 ± 55.66 ms, similar to the vehicle groups (*p* = 0.998), 18.6 mg. L^−1^ (*p* = 0.999), 27.9 mg. L^−1^ (*p* = 0.774), however, were higher than the other treated groups. The group treated with 46.5 mg. L^−1^ (606.3 ± 43.03 ms) was similar to the treated group 37.2 mg. L^−1^ (*p* = 0.914) (Fig. 5F).

The PQ interval during recovery in the control was 96.22 ± 3.56 ms, similar to the vehicle group (*p* = 0.930) the group 18.6 mg. L^−1^ (*p* = 0.363), 27.9 mg. L^−1^ (*p* = 0.519), 37.2 mg. L^−1^ (*p* = 0.295), and 46.5 mg. L^−1^ (*p* = 0.339). The groups treated with 46.5 mg. L^−1^ and 37.2 mg. L^−1^ they were lower than those in the vehicle group (Fig. 5G).

In the recovery period, the duration of the QRS complex for the control group was similar (18.56 ± 1.42 ms) and was similar to the vehicle and all groups treated with TVEO (F (5,48) = 0.860, *p* = 0.514) (Fig. 5H).

In the recovery period, the ST interval for the control group was 308.8 ± 8.85 ms was similar to the vehicle group and the other groups treated with TVEO (F (5,48) = 1.576, *p* = 0.187) (Fig. 5I).

**Fig. 6:**
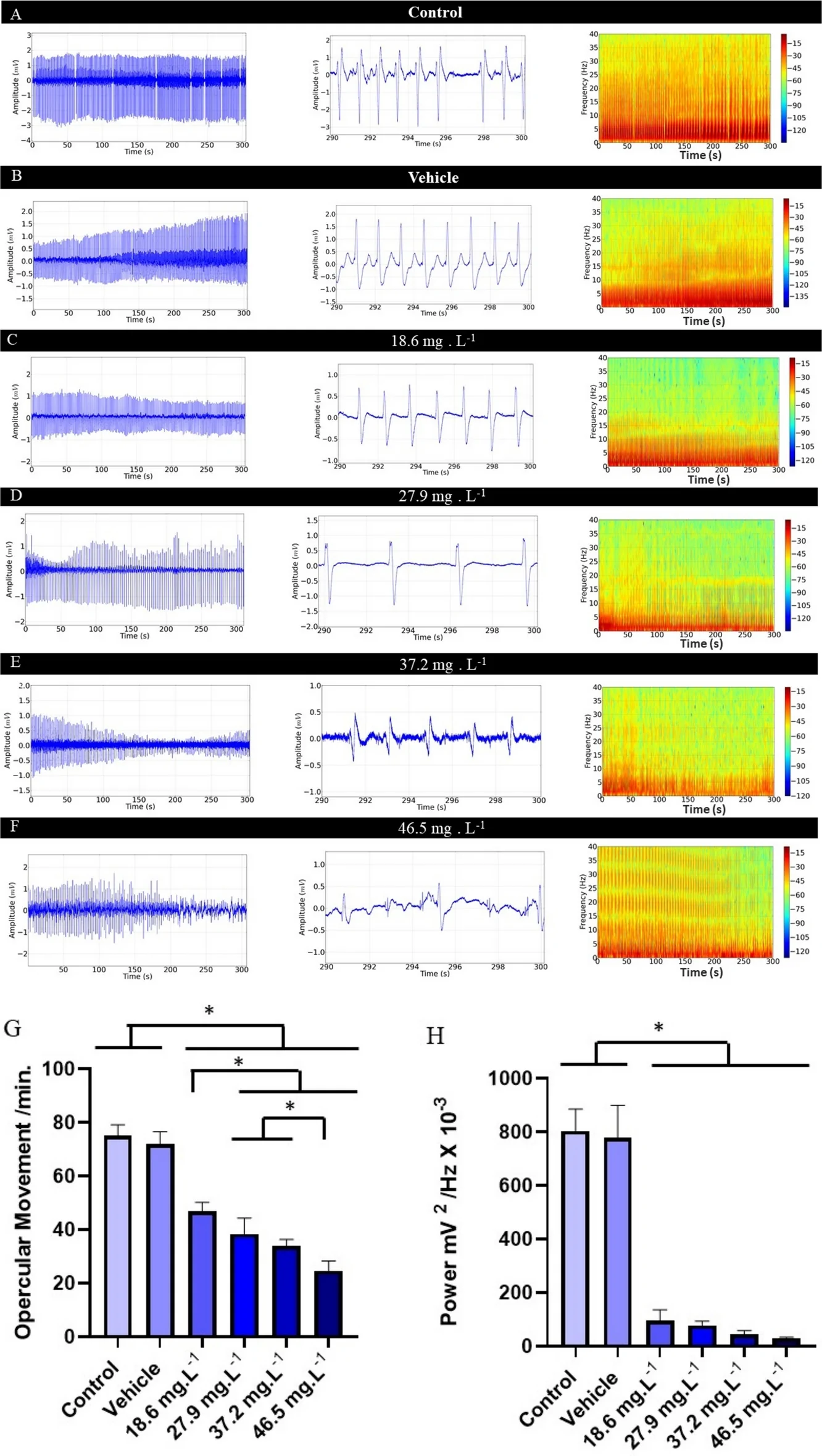
Record of opercular activity in juveniles of *Colossoma macropomum,* undergoing immersion bath treatment at different concentrations of TVEO (left). Amplification of the recording in the last 10 s (290–300 s) (center) demonstrating respiratory activity and spectrogram with energy distribution up to 40 Hz (right). Data obtained for the following groups: control group (**A**); vehicle group (**B**); group treated with 18.6 mg. L^−1^ (**C**); treated with 27.9 mg. L^−1^ (**D**); treated with 37.2 mg. L^−1^ (**E**); and treated with 46.5 mg. L^−1^ (**F**). It shows the average values of opercular motion per minute (omm) (**G**) and mean values of the power of opercular motion (mV^2^/Hz) (**H**). (ANOVA followed by Tukey’s test; ^*^p < 0.05; *n* = 9)

During treatment with TVEO at concentrations of 18.6 mg. L^−1^, 27.9 mg. L^−1^, 37.2 mg. L^−^1, and 46.5 mg. L^−1^ opercular movement frequency (OMM) showed a reduction dependent on the concentration used in the treatment (Fig. 6A–F). The control group had a mean of 75.11 ± 4.01 omm was similar to the vehicle group (*p* = 0.637). However, they were higher than the other groups treated. The group treated with 18.6 mg. L^−1^ (46.89 ± 3.33 omm) was superior to the other treated groups. The group treated with 27.9 mg. L^−1^ (38.44 ± 5.89 omm) was similar to the group treated with 37.2 mg. L^−1^ (*p* = 0.218). However, they were superior to the group treated with 46.5 mg. L^−1^ (Fig. 6 G).

There was a reduction in the power in the records of opercular movements (omm) was observed in the induction of anesthesia, the control group presented a mean power of 804.3 ± 81.38 mV^2^/Hz × 10^–3^, which was similar to the vehicle group (*p* = 0.952), but the other treated groups were higher. The group treated with 18.6 mg. L^−1^ presented a mean (95.98 ± 40.45 mV^2^/Hz × 10^–3^) was similar to the other treated groups, 27.9 mg. L^−1^ (p = 0.990), 37.2 mg. L^−1^ (*p* = 0.536), and 46.5 mg. L^−1^ (*p* = 0.213) (Fig. 6H).

**Fig. 7:**
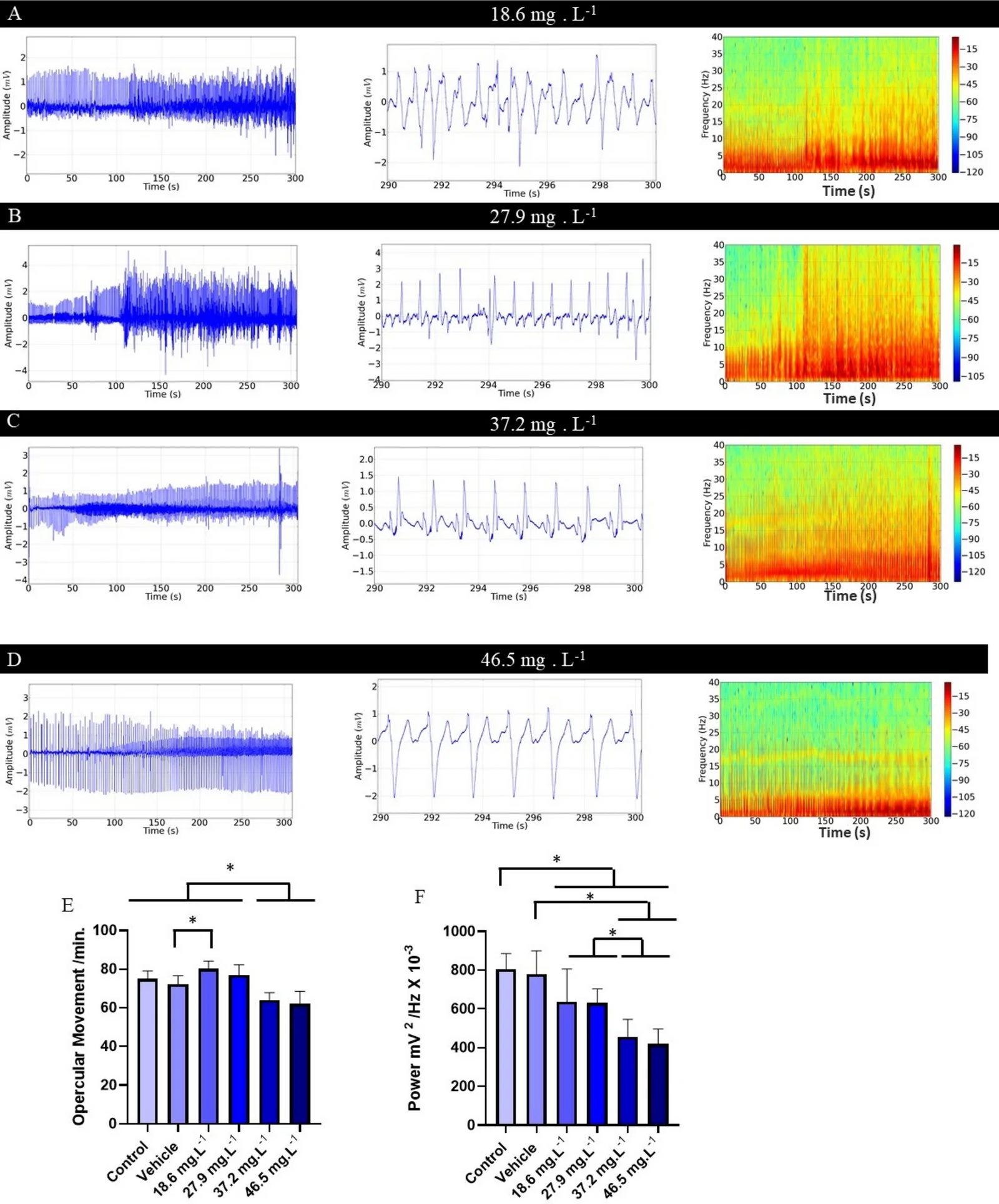
Recording of opercular activity in the recovery period of the 5-min TVEO treatment (left). Amplification of the recording in the last 10 s (290–300 s), to identify opercular movements (center), Spectrogram demonstrating the power intensity up to 40 Hz during recovery after the treatment of TVEO (lasting 5 min) (right). Evaluated in the following treatments: 18.6 mg. L^−1^ (**A**), 27.9 mg. L^−1^ (**B**), 37.2 mg. L^−1^ (**C**), and 46.5 mg. L^−1^ (**D**). Mean values of opercular movement per minute (**E**); mean values of opercular motion power (mV^2^/Hz) (**F**). (ANOVA followed by Tukey’s test; ^*^p < 0.05; *n* = 9)

During the anesthesia recovery period, the opercular movement rate (OMM) showed recovery dependent on the concentration of the treatment (Fig. 7A–D). The control group had a mean of 75.11 ± 4,014 omm was similar to the vehicle groups (*p* = 0.764), group treated with 18.6 mg. L^−1^ (*p* = 0.224), treated with 27.9 mg. L^−1^ (*p* = 0.967), but was higher than the groups treated with 37.2 mg. L^−1^ and 46.5 mg. L^−1^. The groups treated with 46.5 mg. L^−1^ (62.00 ± 6.42 omm) was similar to the group treated with 37.2 mg. L^−1^ (*p* = 0.947) (Fig. 7E).

The increase in potency in the records of opercular movements (omm) was observed in the recovery of anesthetic. The control group had an average power of 804.3 ± 81.38 mV^2^/Hz × 10^–3^, which was similar to the groups in vehicle recovery (*p* = 0.995), but were higher than the other treated groups. The group treated with 18.6 mg. L^−1^ (636.2 ± 169.4 mV2/Hz × 10^–3^) was similar to the vehicle group (*p* = 0.071) and 27.9 mg. L^−1^ (*p* = 0.999). The group treated with 46.5 mg. L^−1^ (420 ± 76.03 mV2/Hz × 10^–3^) was similar to the group treated with 37.2 mg. L^−1^ (*p* = 0.978) (Fig. 7F).

## Discussion

Regarding anesthetic induction and recovery, behavioral analysis indicated efficiency at concentration of 18.6 to 46.5 mg. L^−1^, with latencies to reach lower anesthetic planes for the highest concentrations, showing good muscle relaxation. However, there are no specific studies with *Colossoma macropomum* that address the effects of thyme (*Thymus vulgaris*) essential oil. The studies found with thymol, which is the major substance found in thyme essential oil, were done on *Cyprinus carpio* (common carp) (Yousefi et al. 2018) present similar behavioral results, including increased latency for postural reflex recovery demonstrated concentration dependent. In another study using thymol and linalool (Boaventura et al. 2024) corroborates the results obtained in the present study, since an increase in anesthetic deepening and a decrease in latency for the loss of the postural reflex were also observed in individuals with *Colossoma macropomum.*

Furthermore, another aspect to consider is the relaxation of the muscles responsible for saccadic movements in tambaqui, which indicates profound muscle relaxation in fish subjected to the thyme essential oil immersion bath. While this parameter is already cited in the literature as an indicator of anesthetic induction, its specific application to evaluate myorelaxation during anesthesia requires further investigation (Khan et al. 2000; Souza et al. 2025). Therefore, future studies addressing this response could help establish it as a reliable evaluative criterion for myorelaxation, a highly desirable characteristic in fish anesthesia.

Thirty minutes after treatment with TVEO there were no significant changes in plasma glucose compared to the control group, indicating that the compound does not interfere acutely in glycemic homeostasis, during treatment there was no discomfort that triggered changes triggered by stress. The absence of global differences between the control, vehicle, and treated groups reinforces that there is no robust metabolic effect in this time interval. But the highest concentration of TVEO (46.5 mg. L⁻^1^) has shown a slight increase in blood glucose compared to the vehicle, the values remained similar to those of the control and within physiological limits, suggesting that TVEO has a safe metabolic profile in the short term, without inducing a relevant hyperglycemic effect (Polakof et al. 2012).

An important methodological limitation of this study is the isolated evaluation of plasma glucose, without the inclusion of other physiological parameters capable of elucidating the mechanisms involved in the observed variations. In fish, blood glucose is strongly influenced by the stress response, mainly by the activation of the hypothalamic–pituitary–interrenal axis, with cortisol being the main hormone responsible for stimulating gluconeogenesis and the acute increase in circulating glucose (Tort 2011). Thus, the absence of concomitant measurement of cortisol and metabolites associated with energy metabolism, such as lactate, limits the interpretation of whether the slight elevation in glycemia observed at the highest concentration of TVEO is due to a direct metabolic effect or a secondary response to stress.

However, studies have shown that TVEO or extract is able to significantly reduce serum cortisol and glucose levels in fish subjected to environmental or pharmacological stressors, indicating a modulatory effect of thyme on neuroendocrine response, energy metabolism and oxidative stress (Zadmajid & Mohammadi 2017; Hoseini & Yousefi 2019). In these studies, the reduction of cortisol was associated with improved metabolic homeostasis and attenuation of physiological responses to stress, reinforcing the importance of an integrated approach. This corroborates the blood glucose data obtained in the present study.

The electrocardiographic evaluation of the fish submitted to the treatment shows changes in cardiac activity demonstrating sinus bradycardia dependent on the concentration used, which corroborates the action of other essential oils in tambaquis (Rodrigues Brandão et al. 2022). Previous studies on thymol, ρ-cymene and carvacrol, the most common compounds in the oil used, which have already demonstrated properties related to the cardiovascular system, such as vasorelaxants, antihypertensives, reduction of oxidative stress, and inflammation, in addition to analgesia in several animal examples (Salehi et al. 2018; Patil et al. 2021).

In addition to recognizing the effect, it is necessary to explore the actions of the oil in different concentrations, in the search for an excellent range of effect for the formulation of a therapeutic window for *Colossoma macropomum*. There is a lack of studies that analyze the cardiac effects of thyme essential oil on the species, especially at concentrations used in this work. However, in one study it used higher concentration, 100, 150, 200, and 300 mg. L⁻^1^, found an accentuation in the speed of anesthetic induction, but did not have any mortality observed, (Boaventura et al. 2022) which demonstrates the safety in the use of this essential oil, but depending on the contact time, side effects can be observed, such as prolonged recovery. This is important to understand that in general terms, induction at higher concentration is safe, but it does not necessarily mean that it is adequate for the cardiovascular system, and thus, further studies are needed for the proper application of higher dosages.

In other fish, such as Zebrafish (*Danio rerio*), a decrease in the anesthetic induction time up to 100 mg. L^−1^ was analyzed with the administration of Thymol, and time increase at concentration of 150 and 200 mg. L^−1^, consequently with increased mortality at these higher concentrations, reaching 60% at the 200 mg. L^−1^. In addition, the higher concentration caused a significant decrease in heart rate, (Félix et al. 2025). In Doctor Fish (*Garra rufa*), one study looked at both thymol and carvacrol, in concentration ranging from 50 to 300 mg.L^−1^, and obtained results highly influenced by concentration, both for induction and recovery as well as for behavior—analyzing characteristics such as speed and mobility; dealing with heart rate, they obtained a negative correlation between concentration and dosage only for thymol, in which only concentration of 50, 100, and 150 mg. L^−1^ were observed (Aydın & Orhan 2021).

In this way, the creation of a correct and more effective therapeutic window for anesthetic induction from thyme essential oil depends on the sensitivity of the species used, and *Colossoma macropmum* presented anesthetic induction in concentrations 18.6 to 46.5 mg. L^−1^ presented myorelaxation and adequate anesthetic depth with few electrocardiographic changes.

However, according to studies carried out by Boaventura et al. (2022), Félix et al. (2025) and Aydın and Orhan (2021), point to the effective and safe use for the cardiovascular system of fish between 50 and 100 mg. L^−1^ of thyme essential oil, as well as its active ingredients present in higher concentration.

During the anesthetic recovery phase, fish treated with the highest concentrations (27.9 mg. L^−1^, 37.2 mg. L^−1^, and 46.5 mg. L^−1^) of TVEO showed a significant increase in heart rate, as evidenced by electrocardiographic recordings. This finding suggests the occurrence of a sinus tachycardia effect, characterized by a compensatory response after the withdrawal of the anesthetic agent, a phenomenon widely described in experimental models with fish subjected to natural anesthetics (Reis et al. 2025) and synthetic anesthetics (Rodrigues da Silva et al. 2020). However, this situation did not compromise the recovery of the animals.

Thus, bradycardia induced during anesthesia can be followed by transient tachycardia during recovery, associated with the restoration of metabolic activity and the reactivation of the central nervous system, which can be explained by the activation of compensatory mechanisms. However, substances toxic to fish such as Cunaniol can cause similar conditions (Hamoy et al. 2023), reinforcing the importance of cardiac monitoring during anesthesia and recovery.

This compensatory increase in heart rate may be related to the release of circulating catecholamines, such as adrenaline and noradrenaline, which act on β-adrenergic receptors in the myocardium, promoting an increase in heart rate and contractility (Maciag et al. 2022). This indicates that during anesthesia in deep planes there is a decrease in autonomic activity due to depression of the central nervous system. In humans, during anesthesia, a decrease in myocardial contractility and heart rate is also observed due to the direct action of the anesthetic on the myocardium (Lymperopoulos et al. 2013).

Considering the modulation of cardiac ion channels, especially voltage-gated calcium channels and potassium channels, plays a central role in the recovery of myocardial excitability after anesthesia (Saberian et al. 2025; Joksimovic et al. 2022). In this context, major compounds of TVEO, such as Thymol, p-cymene, and Carvacrol, have pharmacological properties capable of directly or indirectly interfering with cardiac electrophysiological stability (Balahbib et al. 2021; Wang et al. 2020; Mortazavi et al. 2021). However, the effects observed in the present results were pharmacologically reversible.

In the work of Reis et al. (2025), *Colossoma macropomum* was exposed to anesthetic baths containing *Lippia alba* essential oil in concentrations ranging from 80 to 140 μL. L⁻^1^. The authors observed that all concentrations evaluated were able to promote the loss of the postural reflex, followed by reversible recovery. However, electrocardiographic analyses indicated that, at concentrations of 120 and 140 μL. L⁻^1^, bradycardia and atrioventricular block occurred during the anesthetic induction phase. Despite these changes, cardiac activity returned to physiological patterns in all groups during recovery, but this recovery was slower in animals exposed to the highest concentration tested (140 μL. L⁻^1^). Vilhena et al. (2022) evaluated the cardiac response of tambaqui anesthetized with *Piper divaricatum* essential oil, and a marked depression in heart rate was observed at all concentrations, especially in 80 μL. L⁻^1^.

In summary, it was observed that the concentration of 40 μL. L⁻^1^ was sufficient to induce fast, deep, and safe anesthesia in the species, while the concentration of 80 μL. L⁻^1^ caused a greater depression of cardiac function, but with reversibility of effect. Thus, the results of the present study are in line with the literature in indicating that the increase in heart rate observed during recovery represents an adaptive physiological mechanism, aimed at restoring cardiovascular homeostasis after anesthesia-induced depression. However, sinus tachycardia is a feature of recovery from TVEO treatment.

In the study by Cao et al. (2025), Linalool was tested in the anesthetic induction and recovery of the fish *Lateolabrax maculatus* (Chinese sea bass), and deep sedation was verified in the spotted sea bass at a concentration of 20 μL. L⁻^1^. The essential oil of *Lippia sidoides* with the constituents Carvacrol (44.50%) and α-citral (73.56%), in concentration of 10 mg. L⁻^1^ has been shown to be an effective anesthetic for transporting juvenile fish. Previous studies (Rocha et al. 2024) with the species *Rhamdia quelen*, showed that the compounds Thymol and Carvacrol induced anesthesia and muscle contractions at concentrations below 25 mg. L^−1^ in animals. Also, in concentrations of 2 and 5 mg. L^−1^, both thymol and carvacrol showed promising results, demonstrating potential analgesic effects without inducing adverse effects to the behavior of fish larvae of the same species.

Lins et al. (2025), in their results, indicated that the essential oil of *Amyris balsamifera* exerts an effective, reversible anesthetic effect dependent on the concentration of the treatment in *Colossoma macropomum*, evidencing a safe therapeutic window between 20 and 25 μL. L⁻^1^, in which superficial anesthesia, rapid recovery and minimal cardiorespiratory depression are observed. On the other hand, higher concentrations (30–40 μL. L⁻^1^) promoted deep anesthesia, associated with greater cardiovascular impairment and prolonged recovery time, reinforcing the importance of adjusting the concentration according to the desired anesthetic plan. From a physiological point of view, the response to treatment with TVEO may be associated with a lower initial suppression of sympathetic tone and a rapid normalization of cardiac electrical conduction, as evidenced by the recovery of electrocardiographic intervals.

In addition, the antioxidant and anti-inflammatory action of compounds such as thymol, carvacrol, and linalool may contribute to the protection of cardiac tissue and the maintenance of electrophysiological integrity during the post-anesthetic period (An et al. 2021; Mortazavi et al. 2021). Thus, the data reinforce that the concentration of 18.6 to 46.5 mg. L⁻^1^ is inserted in a safe therapeutic window, capable of inducing reversible anesthesia without prolonged impairment of cardiovascular function, which supports its applicability in anesthetic protocols for *Colossoma macropomum*.

Although the opercular beat frequency is performed, the efficiency of the force in the execution of the movement decreased intensely at all concentrations during induction, which indicates that the components of the essential oil may have synergism in the depression of the central nervous system. However, during recovery, the opercular beats showed recovery of the power and frequency of respiratory movements, demonstrating pharmacological reversibility of the effects.

## Conclusion

Thus, it is concluded that essential oil of *Thymus vulgaris* L. presented an effective anesthetic effect and dependent on the concentration in juveniles of *Colossoma macropomum*, promoting loss of postural reflex, immobility and reduction of opercular activity, associated with reversible sinus bradycardia. The electrocardiographic changes observed during induction were transient and did not result in arrhythmias or permanent cardiac impairment. Recovery was complete in all groups, with a more pronounced compensatory response at the highest concentrations. Plasma glucose showed no relevant changes, indicating metabolic stability. The concentration is 18.6 mg. L⁻^1^ was identified with more superficial planes, and concentrations of 27.9, 37.2, and 46.5 mg. L⁻^1^ as a window for anesthesia in moderate to deep planes.

## Data Availability

The authors declare that, in case raw data files in an alternative format are required, these are available upon reasonable request to the corresponding author.
